# Synthetic lethal approaches to target cancers with loss of PTEN function

**DOI:** 10.1016/j.gendis.2022.12.015

**Published:** 2023-11

**Authors:** Ayse Ertay, Rob M Ewing, Yihua Wang

**Affiliations:** a Biological Sciences, Faculty of Environmental and Life Sciences, University of Southampton, Southampton SO17 1BJ, UK; b Institute for Life Sciences, University of Southampton, Southampton SO17 1BJ, UK

**Keywords:** PTEN, Tumour suppressor gene, Cancer, Synthetic lethality, WDHD1

## Abstract

*Phosphatase and tensin homolog* (*PTEN*) is a tumour suppressor gene and has a role in inhibiting the oncogenic AKT signalling pathway by dephosphorylating phosphatidylinositol 3,4,5-triphosphate (PIP_3_) into phosphatidylinositol 4,5-bisphosphate (PIP_2_). The function of PTEN is regulated by different mechanisms and inactive PTEN results in aggressive tumour phenotype and tumorigenesis. Identifying targeted therapies for inactive tumour suppressor genes such as *PTEN* has been challenging as it is difficult to restore the tumour suppressor functions. Therefore, focusing on the downstream signalling pathways to discover a targeted therapy for inactive tumour suppressor genes has highlighted the importance of synthetic lethality studies. This review focuses on the potential synthetic lethality genes discovered in PTEN-inactive cancer types. These discovered genes could be potential targeted therapies for PTEN-inactive cancer types and may improve the treatment response rates for aggressive types of cancer.

## Introduction

### PTEN

*PTEN* deleted on chromosome 10 was identified as a tumour suppressor gene located on the 10q23 chromosome band.^1^ *PTEN*, also known as tensin-like phosphatase 1 (*TEP1*) or mutated in multiple advanced cancers 1 (*MMAC1*) was first identified as a lost or mutated phosphatase in various cancer types such as brain, breast, kidney, and prostate in 1997.^2–4^ *PTEN* is the second most mutated or deleted gene after *TP53* in different cancer types.^5^

At the end of the 1990s and the beginning of the 2000s, both *in vitro* and *in vivo* studies showed that loss of *PTEN* expression contributes to oncogenesis, reduced apoptosis, and increased proliferation and migration of cells.^6–13^ *In vivo* studies showed that PTEN plays a role during embryonic development as loss of *PTEN* contributes to severe hyperproliferation and the failure to elicit apoptosis, causing early embryonic mortality.^6,8,9^ Moreover, heterogeneous deletion of *PTEN* causes carcinogenesis that identified *PTEN* as a haploinsufficient tumour suppressor gene.^1,14^ Wild-type (WT) *PTEN* promotes apoptosis and inhibits cell migration and cell cycle progression.^15,16^ Additionally, PTEN plays a role in activating DNA damage checkpoints to prevent genetic instability.^17^

**Figure 1:**
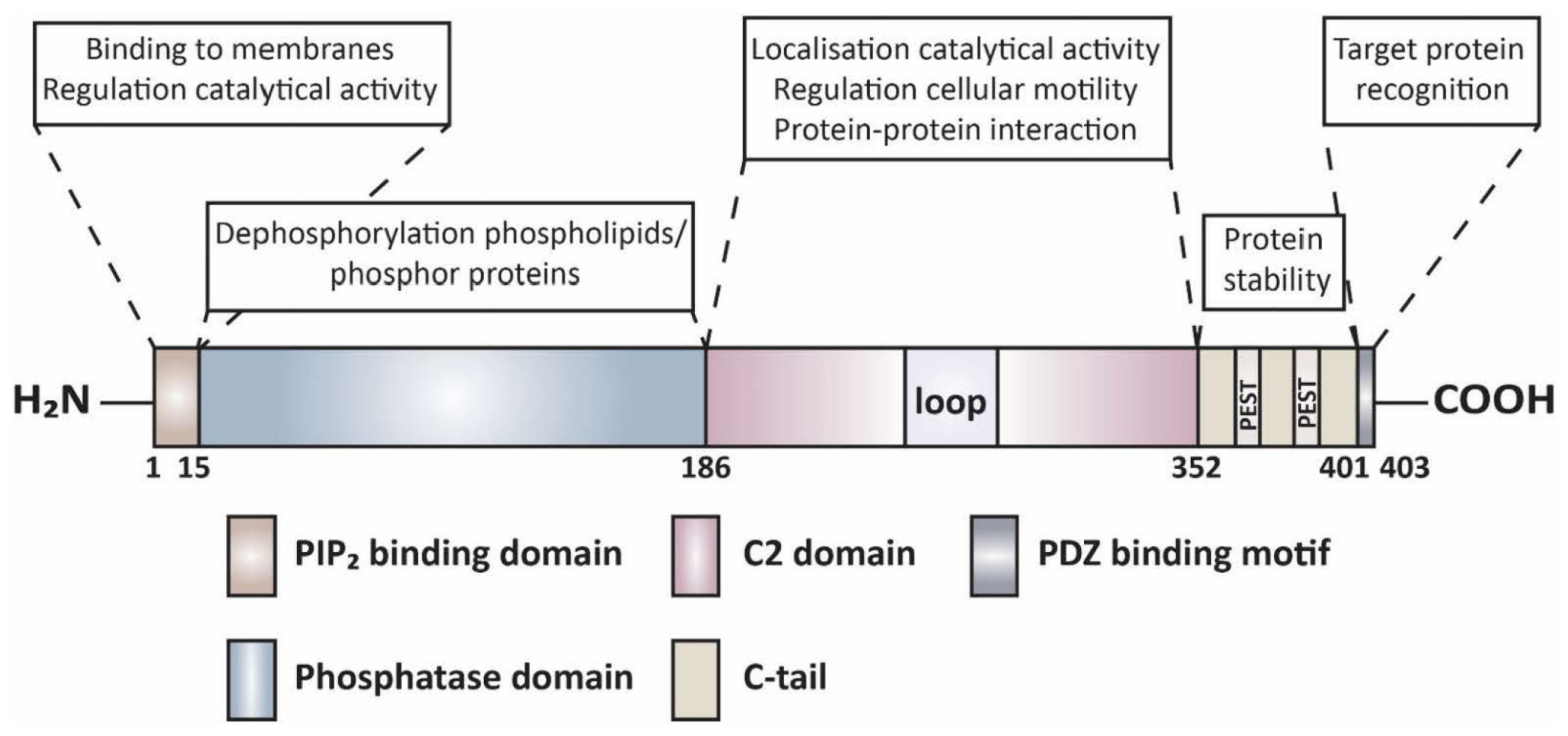
PTEN protein domain structure. PTEN has 403 amino acids and contains five domains; N-terminal PIP_2_ binding domain (residues 6-15), the N-terminal phosphatase domain (15-186), C2 domain (186-352), the C-tail (352-403) and PDZ binding motif. “Loop” represents a conserved but flexible region, from residues 286 to 309 in the C2 domain. The C-tail contains two PEST (proline, glutamic acid, serine, threonine) sequences. The PIP_2_ binding domain has a role to mediate membrane binding and regulate the catalytical activity, the phosphatase domain regulates enzymatic activity, the C2 domain is responsible for cellular localisation and protein-protein interaction, C-tail is responsible for protein stability and PDZ binding motif functions for the recognition of target protein. [Information collected from ^185,186^].

PTEN contains two main active domains; one at the N-terminus and one at the C-terminus (Fig. 1).^18^ The N-terminal domain has lipid phosphatase activity, which is the main domain for the tumour suppressor role of PTEN.^19,20^ The N-terminal domain contains the PIP2 binding domain (PBD) and phosphatase domain, which has an enzymatic and phosphatase-activity role.^21,22^ The C-terminal domain consists of the C2 domain and C-tail region with PDZ motif which is involved in PTEN stability,^23^ and protein-protein interactions.^24^ The C2 domain of PTEN modulates its stability,^23^ and its recruitment to the phospholipid membranes.^25^ Crystal structure analysis of the C2 domain demonstrated a β-sandwich structure, which forms a loop and is involved in DNA and other protein interaction.^18^ Additionally, the C2 domain of PTEN is also involved in the interaction with the centromere.^26^

### PTEN and AKT signalling pathway

PTEN is a dual-specificity phosphatase,^19,27,28^ and its phosphatase activity dephosphorylates phosphorylated tyrosine, serine, and threonine residues in peptide substrates.^28^ PTEN also has lipid phosphatase function as it dephosphorylates PIP3 into PIP2 and inhibits several PIP3-dependent kinases such as PI3K/AKT/mTOR signalling pathway,^19,27^ which is the primary physiological target of PTEN.^29–31^

PI3Ks are a family of intracellular lipid kinases which phosphorylate the 3-position hydroxyl group of the inositol ring of phosphatidylinositol.^32^ PIP_3_ is the primary substrate of PTEN and the catalytic product of PI3Ks.^32^

**Figure 2:**
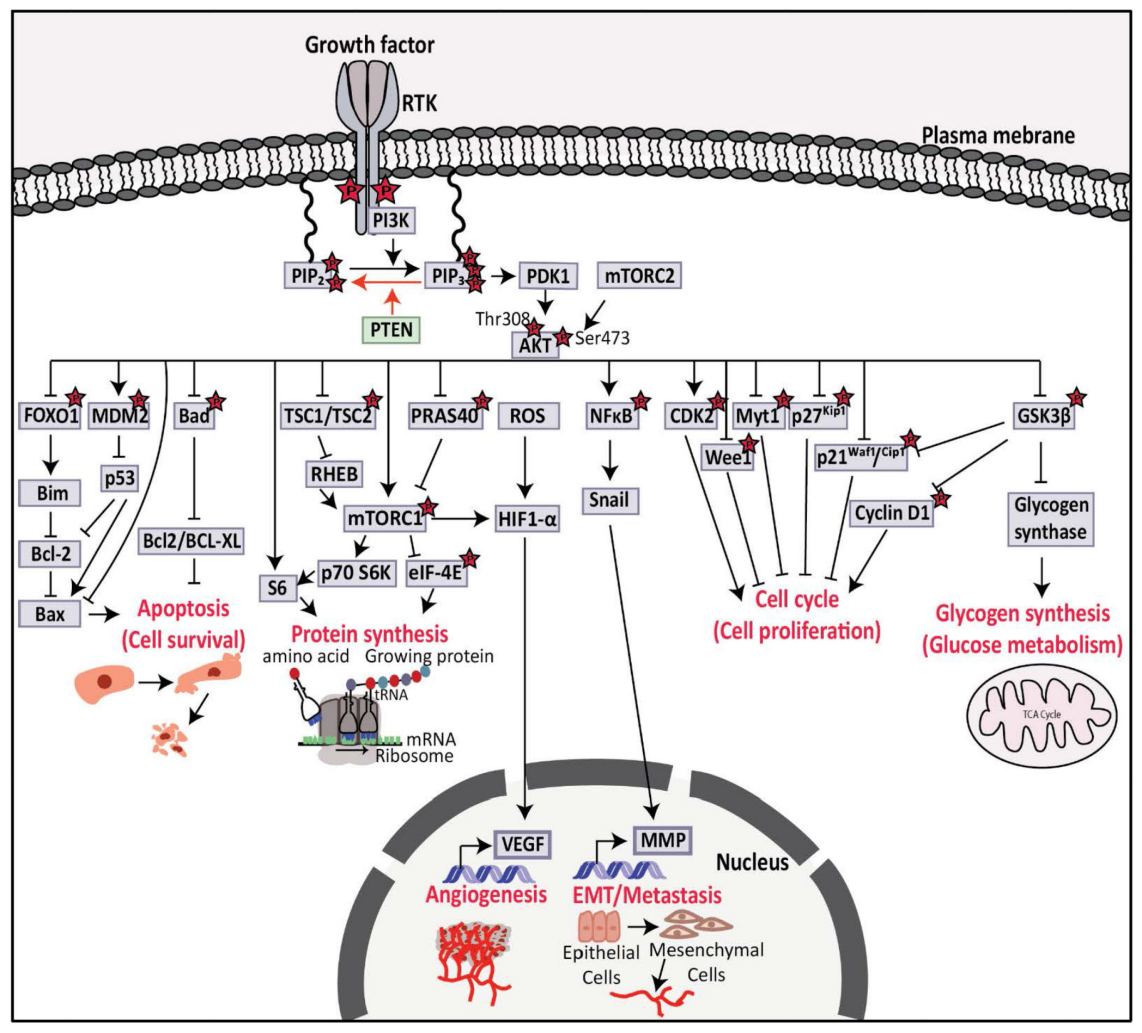
Diagram showing PTEN/PI3K/AKT signalling pathway. Upstream of PI3K/AKT pathway includes RTKs. PTEN suppresses the function of PI3K by dephosphorylating PIP_3_ into PIP_2_ and causes the inactivation of AKT through PDK1. However, loss of *PTEN* activates AKT, which influences its downstream pathways such as inhibition of FOXO1 and Bad and activation of MDM2 to suppress apoptosis. Activation of AKT due to the loss of *PTEN* inhibits TSC1/TSC2 and PRAS40 and activates mTORC1 which leads to protein synthesis. Active AKT also activates NFκB and contributes to EMT; activates CDK2, and inhibits Wee1, Myt1, p27^Kip1^, p21^Waf1/Cip1,^ and GSK3β which leads to cell proliferation. Active AKT also inhibits GSK3β to increase glucose metabolism. PTEN: phosphatase and tensin homolog; PI3K: phosphoinositide 3-kinase; RTK: receptor tyrosine kinase; PIP_2_: phosphatidylinositol 4,5-bisphosphate; PIP_3_: phosphatidylinositol 3,4,5-triphosphate; PDK1: phosphoinositide-dependent kinase 1; FOXO1: forkhead box O1; MDM2: mouse double minute 2 homolog; Bad: B cell lymphoma 2 (BCL-2) antagonist of cell death; TSC1/TSC2: tuberous sclerosis 1/2; PRAS40: proline-rich AKT substrate of 40 kDa; mTORC1: mammalian target of rapamycin complex 2; NFκB: nuclear factor kappa B; EMT: epithelial-mesenchymal transition; CDK2: cyclin-dependent kinase 2; Myt1: Myelin transcription factor 1; GSK3β: Glycogen synthase kinase 3 beta. [Information collected from ^187^].

In the absence or loss of *PTEN*, proteins that contain pleckstrin homology domains such as AKT family members and phosphoinositide-dependent kinase 1 (PDK1), are recruited to and activated on the cell membrane by excessive PIP_3_.^33,34^ AKT isoforms have two residues which are Thr308 and Ser473 and are phosphorylated by PDK1 and mammalian target of rapamycin complex 2 (mTORC2), respectively.^35^ AKT is activated by the phosphorylation of Thr308 and Ser473 residues of AKT.^35^ AKT1, AKT2, and AKT3 are active AKT isoforms and can regulate cell survival, protein synthesis, angiogenesis, epithelial-mesenchymal transition (EMT), metastasis, cell proliferation, and glucose metabolism by phosphorylating downstream signalling proteins (Fig. 2).^35^ Active AKT can also regulate cell survival by inhibiting forkhead box O1 (FOXO1),^36^ B cell lymphoma 2 (BCL-2) antagonist of cell death (Bad),^37^ and activating mouse double minute 2 homolog (MDM2),^38^ Protein synthesis is also regulated by active AKT with the inhibition of tuberous sclerosis 1/2 (TSC1/TSC2),^39^ and proline-rich AKT substrate of 40 kDa (PRAS40),^40^ and activating mammalian target of rapamycin complex 1 (mTORC1),^41^. Moreover, activated mTORC1 and reactive oxygen species (ROS) drive up-regulation of hypoxia-induced factor 1-alpha (HIF1-α) and vascular endothelial growth factors (VEGFs) transcriptional activation to regulate angiogenesis.^42^ Active AKT regulates EMT/metastasis by phosphorylation of nuclear factor kappa B (NFκB),^43,44^ and regulates cell proliferation by phosphorylation of cyclin-dependent kinase 2 (CDK2) and inhibition of Wee1, myelin transcription factor 1 (Myt1), p27^Kip1^, p21^Waf1/cip114^, and glycogen synthase kinase 3 beta (GSK3β).^45^ Additionally, inhibition of GSK3β can also regulate glucose metabolism.^1,46^

## Regulation of *PTEN*

**Figure 3:**
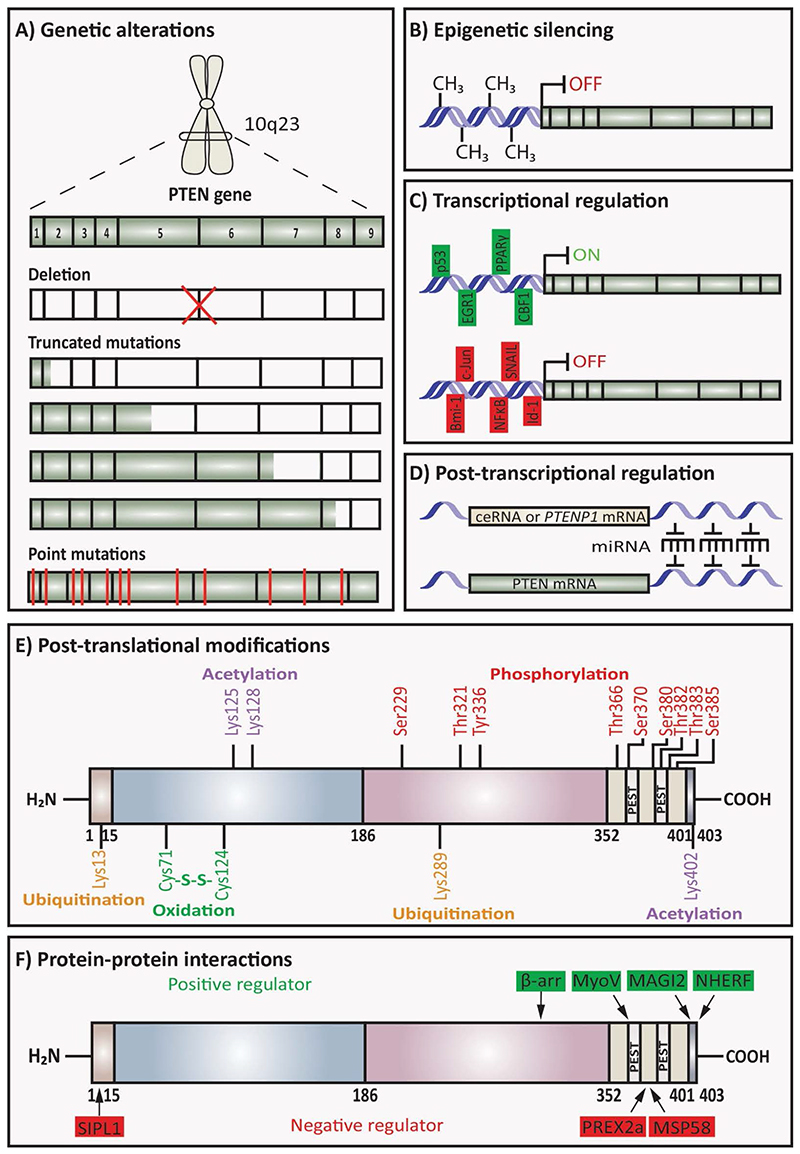
Regulation of PTEN. **A)** Genetic alteration; deletion and mutations of *PTEN* can regulate PTEN expression. **B)** Epigenetic silencing; *PTEN* expression can be silenced by abnormal gene promoter methylation or abnormal modification of histones. **C)** Transcriptional regulation; transcription factors that can bind to *PTEN* promoter are positive or negative regulators of *PTEN* transcription. **D)** Post-transcriptional regulation; miRNAs can regulate *PTEN* expression. **E)** Post-translational modifications; phosphorylation, ubiquitination, oxidation, and acetylation can regulate PTEN. **F)** Protein-protein interactions; interaction of PTEN with proteins can affect PTEN activity. [Information collected from ^1^].

Various molecular mechanisms that regulate PTEN influence the functional PTEN levels in sporadic cancers, inherited syndromes, and other diseases. PTEN is regulated or altered by different mechanisms such as genetic alterations, epigenetic silencing, transcriptional, post-transcriptional regulation, post-translational modifications, and interaction with different proteins, which could initiate and progress cancer (Fig. 3).^1,47^ Therefore, a decrease in PTEN expression causes aggressive tumour phenotype and tumorigenesis in different cancer types.

### Genetic alterations of *PTEN*

Germline and somatic mutations of PTEN including large deletions, intragenic deletions, and insertions, missense, nonsense, and splice site variants can be found in the promoter and all exons of PTEN (Fig. 3A).^48^ Truncated PTEN mutations can be produced by nonsense mutation and lack C-terminal tail and PDZ-binding motif, which play important roles in PTEN protein stability and recruitment to the membrane.^49^

PTEN hamartoma tumour syndromes (PHTS): Cowden syndrome, PTEN-related Proteus syndrome, Bannayan-Riley-Ruvalcaba syndrome, and Proteus-like syndrome are inherited cancer syndromes, which develop due to the *PTEN* germline mutation.^50,51^ Approximately 80% of PHTS patients have *PTEN* germline mutations.^52^ People with PHTS are more prone to develop cancers, such as breast cancer, who have hamartomatous excessive growth in breast tissue,^52^ because the function of PTEN is exerted in the initiation and the progression of cancer.^53^ Almost 70% of PTEN mutations are observed in exon 5, exon 7, and exon 8 in Cowden syndrome, and 40% of these mutations are found in exon 5 which encodes the phosphatase core motif.^52^ Similar results were also observed in another study which showed that 32% of PTEN mutation in Cowden syndrome were observed in exon 5, 13% in exon 7 and 16% in exon 8.^54^ As exon 5 encodes a phosphatase domain, a mutation in exon 5 abrogates the tumour suppressor role of *PTEN*.^55,56^ Moreover, sporadic PTEN mutations are observed in different cancer types such as glioblastoma multiforme (GBM) (19-32%), endometrial (21%), prostate (17-21%), malignant melanoma (14-16%), and breast (4-11%).^57^ However, tumours with PTEN mutations can still have the partial or full catalytic function of PTEN which led to the hypothesis that different mechanisms can inactivate PTEN such as mutation at lysine (Lys, K)289 that change PTEN protein localisation.^58^

### Epigenetic silencing of PTEN

In different cancer types, abnormal gene promoter methylation or abnormal modification of histones causes epigenetic silencing of *PTEN* expression (Fig. 3B). Hypermethylation of CpG islands in the *PTEN* promoter can silence the transcription of *PTEN* in breast cancer and melanoma.^59,60^ Sal-like protein 4 (SALL4), a zinc-finger transcription factor, recruits an epigenetic repressor complex (Mi-2/NuRD) that contains ATP-dependent nucleosome remodelling activity and a histone deacetylase to the *PTEN* locus and leads to condensed heterochromatin and represses *PTEN* expression.^61^ Despite the *PTEN* mutation frequency being low in breast cancer, the frequency of *PTEN* promoter methylation is 50% in breast cancer cases.^62^ Thus, epigenetic silencing of PTEN inactivates this tumour suppressor gene and leads to activation of oncogenic AKT signalling.^59^

### Transcriptional regulation of PTEN

Different transcription factors have binding sites at the *PTEN* promoter and are known as positive or negative regulators of *PTEN* transcription (Fig. 3C).

There is a p53 binding site at upstream of the *PTEN* gene and it was shown that p53 induction in primary and tumour cell lines with WT p53 upregulates *PTEN* mRNA levels compared to mutant p53 cells.^63^ Early growth regulated transcription factor 1 (EGR1), peroxisome proliferator-activated receptor gamma (PPARγ) and C-repeat binding factor 1 (CBF1) also upregulate the expression of *PTEN*. It has been shown that EGR1 binds to the *PTEN* promoter and due to the stimulation of insulin-like growth factor 2 (IGF-2) by a negative-feedback loop,^64^ *PTEN* expression is upregulated. Activated PPARγ can also bind to the *PTEN* promoter and this leads to the up-regulation of *PTEN* in both normal and cancerous cells such as macrophages, colorectal cancer cells, and breast cancer cells,^65^ For example, PTEN expression increases with rosiglitazone (PPARγ selective ligand to activate PPARγ) and this decreases hepatocarcinoma cell line (BEL-7404) migration.^66^ Moreover, it has been shown that transcriptional levels of *PTEN* are regulated by the Notch-1 signalling pathway via the CBF-1 transcription factor which binds to the minimal *PTEN* promoter.^67^

On the other hand, mitogen-activated protein kinase kinase 4 (MKK4) is a negative regulator of *PTEN* transcription that works by activating NFκB that binds to the *PTEN* promoter region.^68^ It has also been shown that transforming growth factor beta (TFGβ) inhibits *PTEN* transcription in mesangial,^69^ and pancreatic cancer cells.^70^ Additionally, c-Jun which is a transcription factor also decreases *PTEN* expression via the binding to the *PTEN* promoter at the variant AP-1 site (PF-1) and the negative correlation between c-Jun and PTEN levels was observed in different human cancer cell lines.^71^ Inhibitor of differentiation-1 (Id-1),^72^ B lymphoma Mo-MLV insertion region 1 homolog (Bmi-1),^73^ and SNAIL,^74^ can also bind to *PTEN* promoter and inhibit its transcription.

These studies indicated that transcriptional control of *PTEN* plays an important role at the intersection of pathways to regulate PTEN expression and has an influence on tumour suppression and tumour promotion.

### Post-transcriptional regulation of PTEN

miRNAs are small non-coding RNA molecules, which have 20-25 nucleotides and regulate gene expression in many cancer types (Fig. 3D). Different studies showed that various miRNAs downregulate the expression of *PTEN* and this can lead to carcinogenesis and metabolic disorders.^75^ miR-21 is one of the oncogenic miRNAs that downregulate the expression of *PTEN* in ovarian, hepatocellular, and lung cancers.^76,77^ Additionally, miR-25 crosslinks the MEK/ERK and PTEN/PI3K/AKT/mTOR signalling pathways because activated ERK increases the expression of miR-25 which then inhibits PTEN protein level and leads to activation of AKT signalling.^78,79^ PTEN expression is also downregulated by *MYC* oncogene via increased expression of miR-19.^80^ PTEN pseudogene 1 (*PTENP1*) and *PTEN* mRNA have significant sequence identity and it has been found that *PTENP1* miR target sites regulate the expression of PTEN via sequestration of PTEN-targeting miR which leads to an increase of *PTEN* mRNA half-life and PTEN protein levels.^81^

### Post-translational modification of PTEN

The role of PTEN is also regulated by post-translational modifications such as phosphorylation, ubiquitination, oxidation, and acetylation (Fig. 3E).

Phosphorylation of PTEN has an impact on PTEN stability, activity, and cellular localisation. Phosphorylation of PTEN on Ser370, Ser380, Thr382, Thr383, and Ser385 is mediated by the protein kinase casein kinase 2 (CK2). The phosphorylation by CK2 leads to the stabilisation of PTEN and closed PTEN conformation that reduces the interaction between the binding partners and decreases its plasma membrane localisation, thus reducing its phosphatase activity.^82,83^ As the phosphorylation in the C-terminal tail stabilises PTEN conformation, this leads to the reduction of interaction with membrane phospholipids or PDZ domain-containing proteins; membrane-associated guanylate kinase inverted 2 (MAGI2), and therefore inhibits its PIP_3_ phosphatase activity.^84^ The inactivation of PTEN can also be seen when PTEN is phosphorylated on Ser385 by LKB1.^85^ The phosphatase activity of PTEN is also reduced with the phosphorylation of PTEN by GSK3β at Thr366.^86^ Additionally, the C2 domain of PTEN is phosphorylated by tyrosine protein kinase RAK at Tyr336,^87^ and RHOA-associated protein kinase (ROCK) at Ser299 and Thr321.^88^

Ubiquitination also regulates PTEN subcellular localisation, vesicle trafficking, and activation. Lys13 and Lys289 are PTEN ubiquitination and monoubiquitination sites and have a role in PTEN cytoplasmic-nuclear shuttling.^58^ Ubiquitin/proteasome pathway can regulate the function of PTEN.^58^ NEDD4-1 is an E3 ubiquitin-protein ligase that can polyubiquitinate PTEN at Lys13 and Lys289 leading to its degradation or it can also monoubiquitinate PTEN at Lys13 and Lys289 regulating its cytoplasmic-nuclear shuttling.^89^ In non-small-cell lung cancer, PTEN is downregulated due to the ubiquitin-mediated degradation by NEDD4-1 and this leads to PTEN activity loss.^90^

Acetylation is another mechanism that regulates PTEN function. The catalytic activity of PTEN is reduced by acetylation at Lys125 and Lys128 by acetyltransferase P300/CREB-binding protein (CBP)-associated factor (PCAF) and at Lys402 by CBP.^91^ ROS are also responsible for regulating PTEN catalytic activity by the oxidative-stress-induced formation of the disulphide bond between active Cys71 and Cys124.^92^

### Protein-protein interactions of PTEN

Many different studies demonstrated that protein-protein interactions also play an important role in PTEN activity due to the effect on its stability, conformation, lipid membrane-binding potential, and subcellular localisation (Fig. 3F).

NA^+^/H^+^ exchanger regulatory factor (NHERF) interacts and recruits PTEN to platelet-derived growth factor receptor (PDGFR) and prevents the activation of the PI3K/AKT signalling pathway.^93^ MAGI2 and β-arrestins interact with PTEN and increase its lipid phosphatase activity to suppress AKT activation.^94,95^ PTEN also directly interacts with motor protein myosin V that leads to the movement of PTEN to the membrane and PTEN can dephosphorylate PIP_3_ into PIP_2_.^96^

Different proteins interact with PTEN and negatively affect its tumour suppressor activity. The oncoprotein MSP58 interacts with PTEN at the C-terminus region leading to cellular transformation.^97^ Parkinson protein 7 (PARK7 also known as DJ1) directly binds to PTEN in oxidative conditions, inhibits PTEN activity, and increases AKT activity, leading to cell proliferation and transformation.^98^ PIP_3_-dependent RAC exchanger factor 2a (PREX2a),^99^ shank-interacting protein-like 1 (SIPL1),^100^ and α-mannosidase 2C1 (MAN2C1),^101^ can also interact with PTEN and directly inhibit its lipid phosphatase activity to convert PIP_3_ into PIP_2_.

## Function of PTEN

PTEN regulates PI3K/AKT signalling with its phosphatase-dependent activity. However, PTEN also has phosphatase-independent functions.^1^ As PTEN can shuttle between the cytoplasm and nucleus, it is a tumour suppressor gene both in the cytoplasm and nucleus.^102,103^

### PTEN and cell metabolism

Metabolic reprogramming leads to rapid cell proliferation. Cancer cells or rapidly proliferating cells convert glucose into lactate via aerobic glycolysis, regardless of the presence of oxygen and this is known as the Warburg effect.^104^ Cellular mediators of signal transduction and gene expression; PTEN/PI3K/AKT/mTOR pathway, HIF1-α, and MYC can affect the metabolic pathways during cell proliferation and carcinogenesis.^105^ PI3K/AKT regulates glucose uptake, and HIF1-α and MYC regulate genes that are involved to regulate glucose and glutamine metabolism.^105^

It has been shown that overexpressing *PTEN* in transgenic mice decreased body size due to the reduction of cell number, increased energy expenditure, and decreased accumulation of body fat.^106^ Additionally, reduction in the glucose and glutamine uptake increased mitochondrial oxidative phosphorylation, and resistance to oncogenic transformation was observed in transgenic mice cells with *PTEN* overexpression.^106^ Another study showed that additional genomic copies of *PTEN* in transgenic mice prevent metabolic pathologies and cancer.^107^

PTEN/PI3K/AKT/mTOR pathway has an important role in regulating glucose metabolism. As PTEN modulates insulin signalling, it has a role in regulating glucose uptake.^108,109^ It has been discovered that overexpression of PTEN in adipocytes reduced the uptake of glucose due to the inhibition of insulin-stimulated, PI3K activation-dependent 2-deoxyglucose uptake and glucose transporter 4 (GLUT4) translocation which is a key event in insulin signalling.^110^ Another study showed that GLUT4 translocation and insulin metabolic function cannot be modulated by PTEN in normal physiological conditions.^111^ However, it was found that PTEN regulates GLUT1 expression and thus glucose uptake in transformed cells such as thyroid cancer cells.^112^ PTEN regulates FOXO, PPAR gamma-coactivator 1 alpha (PGC1α), and inhibits gluconeogenesis.^107^ Moreover, PI3K/AKT signalling inhibits GSK3, which activates lipogenic transcription factor sterol-regulatory element-binding protein 1C (SREBP1C), thus loss of *PTEN* induces adipogenic-like transformation and genes involved in lipogenesis and β-oxidation via PPARγ and SREBP1C.^113^

### PTEN and cell motility/polarity

PTEN/PI3K signalling pathway has been shown to have a role in migration both in development and cancer cells. Genetic deletion of *PTEN* in mouse fibroblast lines induced cell motility via overexpression of key downstream effectors of the PI3K pathway: RAC1 and CDC42, which promote cell migration.^11^ The migration of glioma cells can be inhibited by the C2 domain of PTEN showing its lipid phosphatase-independent activity,^114^ which may indicate the influence of the PI3K pathway-independent effect of PTEN.^115^ It has also been shown that glioblastoma cell migration was enhanced with the knockdown of *PTEN* via focal adhesion kinase (FAK). FAK is a cytoplasmic phosphoprotein and is activated by integrins which can be dephosphorylated by PTEN, inhibiting the migration of cells.^116^ Moreover, SHC is also dephosphorylated by PTEN and this inhibits downstream MAPK that has a role in cell motility.^117^

To establish the polarity of the cell, when PTEN is found on the apical cell membrane during epithelial morphogenesis, PTEN and PIP_2_ recruit annexin 2 (ANXA2), CDC42 and partitioning defective 6 (PAR6)-atypical protein kinase C (aPKC) to the apical plasma membrane.^118^ Therefore, the normal development of the apical surface and lumen might be blocked with the loss of *PTEN* and could lead to changing of cells from epithelial to mesenchymal properties and increase the cell motility and invasion which is known as EMT.^73^

### PTEN and tumour microenvironment

The role of PTEN in regulating the tumour microenvironment has also been identified. The tumour microenvironment consists of immune and stromal cells.^119^ Loss of *PTEN* activity not only affects the cancer cell behaviour but also affects the tumour microenvironment and immune-infiltrate composition. Studies showed that loss of *PTEN* function leads to tumour microenvironment remodelling and formation of immunosuppressive tumour microenvironment with properties such as reduced frequency of cytotoxic T cells, helper T cells, and natural killer (NK) cells, increased levels of pro-oncogenic inflammatory cytokines and increased frequency of immunosuppressive cells.^120–122^

Genetic and epigenetic changes in *TP53* and *PTEN* were observed in stromal fibroblasts from the tumour microenvironment of human breast cancer samples.^123^ Stromal fibroblasts are the major important cell types that can shape the microenvironment architecture leading to tumour growth and progression.^124^ Trimboli *et al*., 2009 showed that the deletion of PTEN in fibroblasts of mouse mammary gland tumours forms a tumour-permissive stroma including remodelling of extracellular matrix and increased collagen deposition, innate immune cell infiltration, and angiogenesis. These features increase the tumour initiation, progression, and malignant transformation of mammary epithelial tumours.^125^ Mechanistically, down-regulation of miR-320 upregulates v-ets erythroblastosis virus E26 oncogene homolog 2 (ETS2) in *PTEN*-deleted mammary stromal fibroblasts which activates an oncogenic secretome that reprogrammes the tumour microenvironment and promotes angiogenesis and tumour cell invasion.^126^ Thus, PTEN regulates the communication between various cellular compartments in the tumour microenvironment, which can influence the cancer phenotype.

### PTEN and angiogenesis

PTEN/PI3K/AKT signalling is also important for angiogenesis via mechanisms such as HIF1-α and transcriptional activation of VEGF.^127^ For example it has been shown that PTEN negatively regulates transcription factor HIF1-α and VEGF and inhibits tumour angiogenesis.^128^ Moreover, overexpressing *PTEN* in a *PTEN*-deficient glioma model significantly reduced tumour growth *in vivo* and increased mice survival which was due to the induction of a negative regulator of angiogenesis, thrombospondin-1 which led to decreased blood vessel formation in the tumour.^129^

### Nuclear PTEN

It has been shown that PTEN can shuttle from the cytoplasm to the nucleus and has a functional role in the nucleus. Thus, *PTEN* is also a tumour suppressor gene in the nucleus and nuclear loss of *PTEN* contributes to more aggressive cancers and can be used as a prognostic marker.^130,131^

**Figure 4:**
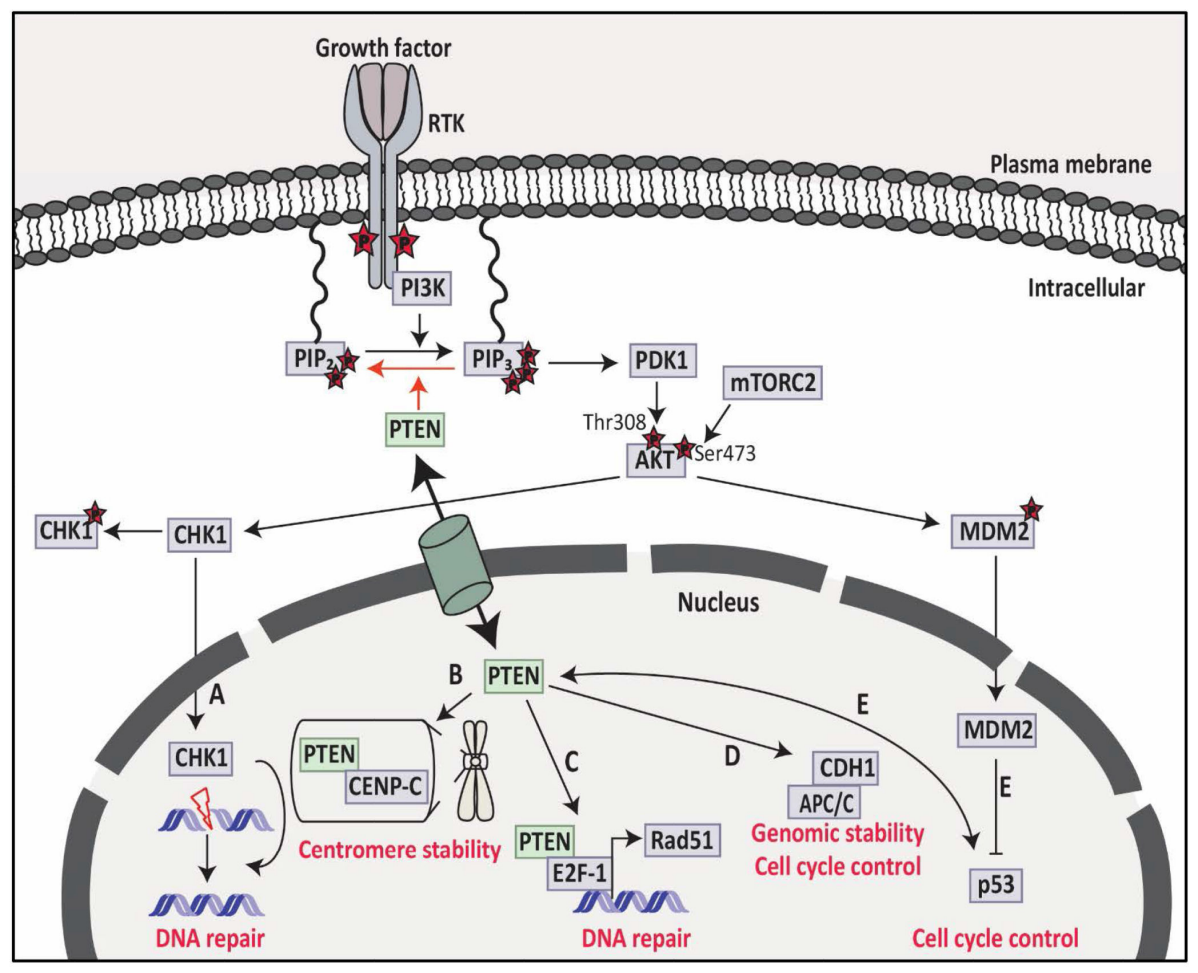
Nuclear functions of PTEN. PTEN has functions both in the cytoplasm and nucleus. **A)** Cytoplasmic PTEN dephosphorylates PIP_3_ into PIP_2_ and inhibits AKT activity and CHK1 phosphorylation, leading to CHK1 translocation into the nucleus for DNA repair. **B)** In the nucleus, PTEN can bind to CENP-C and maintain centromere stability. **C)** PTEN interacts with E2F-1 and leads to transcriptional regulation of Rad51 to control DNA repair in the nucleus. **D)** Nuclear PTEN enhances the interaction between APC/C and CDH1 to maintain genomic stability and control the cell cycle. **E)** Nuclear PTEN interacts with p53 to control the cell cycle due to the phosphatase-dependent and phosphatase-independent activities of PTEN. PTEN: phosphatase and tensin homolog; PIP_2_: phosphatidylinositol 4,5-bisphosphate; PIP_3_: phosphatidylinositol 3,4,5- triphosphate; CHK1: checkpoint kinase 1; CENP-C: centromere protein-C; APC/C: anaphase-promoting complex/cyclosome; CDH1: CDC20 homologue 1. [Information collected from ^1^].

Puc *et al*., 2005 discovered that loss of *PTEN* promotes genomic instability in tumours via checkpoint kinase 1 (CHK1), which is involved in cell cycle progression and this reflects one of the PTEN functions in the nucleus.^17,132^ Mechanistically, when *PTEN* is deficient, the cytoplasmic AKT signalling pathway is activated and contributes to CHK1 degradation by phosphorylation and subsequent ubiquitination in the cytoplasm and entry of CHK1 to the nucleus is prevented (Fig. 4A).^132^ Both *in vitro* (in embryonic stem cells),^17^ and *in vivo* (primary breast carcinoma),^132^ studies demonstrated that *PTEN* deficiency leads to the accumulation of unrepaired double-strand breaks due to the lack of CHK1, in G2 checkpoint and stimulates tumour development. Apart from PTEN/AKT/CHK1 mechanism, nuclear PTEN has two other mechanisms related to its tumour suppressive role to maintain chromosomal stability.^1,26^ First, PTEN interacts with centromeres by physical association with integral kinetochore component centromere protein-C (CENP-C) (Fig. 4B). The physical interaction between PTEN and CENP-C does not require PTEN phosphatase activity as PTEN with the PTENC124S mutation was able to interact with CENP-C.^26^ However, a specific nonsense mutation, R189X, of PTEN which lacks the entire C-terminus but has the intact N-terminal phosphatase domain showed a disruption of the interaction and led to centromere breakage and chromosomal translocations.^26^ Secondly, PTEN could be essential for DNA repair as PTEN null-type cells showed DNA double-strand breaks. It has been shown that PTEN interacts with the E2F-1 transcription factor to regulate the key component for homologous recombination repair of DNA double-strand breaks (Rad51) but *PTEN* deficiency prevents this interaction (Fig. 4C).^26^ Similar to the first mechanism described above, Rad51 regulation was PTEN phosphatase activity independent because the PTENC124S mutant, lacking catalytical activity did not change Rad51 expression.^26^

The phosphatase-independent activity of PTEN increases the E3-ligase activity of anaphase-promoting complex/cyclosome (APC/C) via the association of APC/C with its activator CDC20 and CDH1 (Fig. 4D).^133^ APC/C-CDH1 complex has tumour suppressive activity which causes the degradation of oncoproteins such as Aurora kinases (AURKs) and polo-like kinase 1 (PLK1).^133,134^ This indicates the phosphatase-independent tumour suppressive activity of nuclear PTEN due to the activation of APC/C-CDH1.^133^

It has also been reported that the role of nuclear PTEN might be regulated by the physical interaction of PTEN with other nuclear target proteins such as p53.^135^ The crosstalk between PTEN and p53 was discovered in mice in which it was showed that loss of *PTEN* causes p53-driven carcinogenesis due to the phosphatase-dependent and phosphatase-independent activities of PTEN.^135^ Loss of *PTEN* activates AKT signalling, phosphorylates MDM2 and translocates MDM2 to the nucleus, which then leads to p53 degradation (Fig. 4E).^38,136^ Thus, PTEN is involved in the stabilisation and transcriptional activity of p53, which has an important function in tumorigenesis.^135^

## Targeting PTEN-inactive cancers

### PI3K/AKT/mTOR pathway

The loss of PTEN function activates the PI3K/AKT/mTOR pathway and results in the growth, proliferation, and survival of cancer cells.^19,27^ Therefore, there have been different studies to target this pathway.

Since the loss of PTEN causes resistance mechanisms to the treatments, pre-clinical studies have been focusing on combination treatments. For example; a recent study that combined the treatment of G-protein-coupled receptor (PAR1), EGFR signalling, and PI3Kβ inhibitor,^137^ and another study combined PI3Kβ inhibitor with paclitaxel (a chemotherapeutic agent) and anti-PD1,^138^ suggested that these combinations could be new potential therapeutic strategies for PTEN-inactive triple-negative breast cancer (TNBC).

The LOTUS trial is one of the phase 2 clinical trials, which showed that the median overall survival in paclitaxel (chemotherapeutic drug) with ipatasertib (AKT inhibitor) arm versus paclitaxel with placebo arm is 23.1 vs 15.8 months in the PTEN low population.^139^ There are also active clinical trials that include combination treatments for PTEN-inactive cancer types such as (1) the combination of PI3K-Beta inhibitor, AZD8186 and chemotherapeutic drug, Docetaxel (NCT03218826), (2) the combination of receptor tyrosine kinase inhibitor, Pazopanib and mTOR inhibitor, Everolimus (NCT01430572), and (3) the combination of PI3K-Beta inhibitor, GSK2636771 and immunotherapy, Pembrolizumab (NCT03131908).

Thus, studies were focused to treat PTEN-inactive cancer by targeting selected components of the PI3K/AKT/mTOR signalling pathway.

### Synthetic lethality

Discovering an effective treatment is challenging due to the genetic abnormalities in cancer cells. Targeting and inhibiting the function of activated ‘druggable’ oncogenes has been successful. For instance, the function of amplified human epidermal growth factor receptor 2 (HER2) is inhibited by the monoclonal antibody, trastuzumab.^140^ Loss-of-function mutations in tumour suppressor genes are major genetic alterations leading to more challenges to identify targeted drugs since it is difficult to restore their functions.^141^ Therefore, studies have been focusing to target downstream signalling pathways that are altered by the inactivation of tumour suppressor genes.^141,142^ This paves the way for studies to focus on a different approach, which is synthetic lethality.

**Figure 5:**
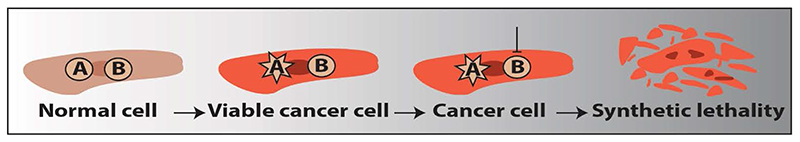
The principle of synthetic lethality. The survival of cancer cells with inactive tumour suppressor gene A (loss of function) depends on the expression of gene B. Inhibition of gene B leads to synthetic lethality (cell death). The star represents the inactive gene. [Information collected from ^146^].

Synthetic lethality is a phenomenon between two genes when the alteration (a mutation, RNAi knockdown, or inhibition) of one gene is viable but the alteration of both genes simultaneously leads to loss of viability (Fig. 5).^143^ Synthetic lethality is an important approach in cancer research since it can be used to target cancers with inactive tumour suppressor genes.^144^

Targeting synthetic lethality provides an alternative approach to cancer treatment.^145,146^ To identify novel targeted therapies, synthetic lethality screens can be performed, including RNA interference (RNAi) screens.^142,147^ One of the well-known examples of synthetic lethality interaction is between *BRCA1/2* and PARP1. *BRCA1/2* are tumour suppressor genes that have a role in homologous-recombination-mediated DNA repair and PARP1 is involved in base excision repair. Tumours with *BRCA1/2* deficiency depend on PARP1 for DNA repair. Thus, inhibition of PARP1 kills *BRCA1/2* deficient tumours.^148,149^

### Synthetic lethality genes/interactions in PTEN-inactive cancer types

As PTEN is the second most mutated gene following *TP53* in different cancer types,^5^ various studies have been performed to identify PTEN synthetic lethal interactions in a variety of cancer types (Table 1). Although, there are no clinical trials for PTEN synthetic lethality yet, discovering PTEN synthetic lethal interactions in cancer may provide potential biomarkers or targeted therapies for the cancer types, which do not have successful treatment options.

#### Poly-ADP ribose polymerase (PARP)

In addition to the synthetic lethality interaction between *BRCA1/2* and PARP1, Christopher J Lord and Alan Ashworth’s group showed the benefits of treatment of *PTEN*-deficient tumours with PARP1 inhibitor and thereby identified the synthetic lethality relationship between *PTEN* and PARP.^150^ They showed that *PTEN*-deficient cancer cells decreased the expression of RAD51, which is involved in homologous recombinant (HR)-mediated DNA repair and this increased the sensitivity to PARP inhibitors.^150^ Moreover, GBM cancer cells treated with temozolomide with PARP inhibitors showed resistance due to the upregulation of HR.^151^ Following studies by another group showed that *PTEN*-deficient GBM patients, which have downregulated HR, can benefit from the combination of PARP inhibitors with the standard treatment of GBM, which is the combination of ionizing radiation and temozolomide.^152^ These studies highlighted the promising treatment option of using PARP inhibitors for *PTEN*-deficient cancer types.

#### MPS1, Mono Polar Spindle 1 (TTK)

Since targeting the identified critical genes could be challenging, Christopher J Lord and Alan Ashworth’s group conducted the first attempt to identify potential ‘druggable’ genes in different breast tumour cell line models by using siRNA targeting the kinome.^153^ It was discovered that *PTEN*-deficient breast tumour cells have a dependency on the gene, *TTK* protein kinase gene that has a role in the mitotic spindle assembly checkpoint. Inhibition of *TTK* by both siRNA and chemically in *PTEN*-deficient cells indicated a novel treatment strategy for PTEN mutant tumours. Mechanistically, the synthetic lethality interaction between *PTEN* and *TTK* was shown as *TTK* inhibition increased the aneuploidy or genomic instability and leads to *PTEN*-deficient selective cell death.

#### Polo-like kinase 1 (PLK1)

One of the previous studies showed that PTEN regulates E3-ubiquitin ligase APC/CDH1, which then causes the degradation of oncoprotein, PLK1.^133^ It was then found that PLK1 expression was increased in *PTEN*-deficient prostate cancer cells, which leads to the adaptation of cells to mitotic stress for survival.^134^ This study discovered that inhibition of *PLK1* could be a potential treatment option for prostate cancer patients with *PTEN* deficiency.^134^

#### Nemo-like kinase (NLK)

Another study from Christopher J Lord and Alan Ashworth’s group also discovered different synthetic lethal genes with PTEN.^154^ By performing RNAi screening in *PTEN*-deficient isogenic models, they identified that *NLK* inhibition could be synthetic lethality. It is known that *PTEN*-deficient cells increase the activation of AKT, which then phosphorylates the tumour suppressor gene, *FOXO1*, and leads to its degradation. Additionally, NLK is known to inactivate FOXO1 via AKT-independent phosphorylation. In this study, it was discovered that *PTEN* and *NLK* synthetic lethality is *FOXO1* dependent. Inhibition of *NLK* increased the nuclear FOXO1 localisation and induced senescence in *PTEN*-deficient cells but not in *PTEN*-proficient cells.^154^

#### Polynucleotide kinase-phosphatase (PNKP)

Protein PNKP, which is an enzyme that has a role in repairing DNA strand breaks was another identified synthetic lethal partner with *PTEN*.^155^ The initial studies showed that PNKP inhibition in *PTEN*-deficient cells sensitized the cancer cells to ionizing radiation.^155–157^

#### Apurinic/apyrimidinic endonuclease (APE1)

APE1 is another protein that has function in DNA base excision repair (BER) and the synthetic lethal link between PTEN and APE1 was identified in melanoma.^158^ Abbotts *et al*., 2014 demonstrated that *PTEN*-deficient cells have defective gene expressions which play a role in DNA double-strand break (DSB) break compared to the *PTEN*-proficient cells. Since the sensitivity, accumulation of DSBs, and apoptosis were increased post-treatment of *APE1* inhibitors, the synthetic lethality relation between *PTEN* and *APE1* was supported in melanoma.^158^ This study showed that blocking BER by *APE* inhibition could be potential targeted therapy for *PTEN*-deficient melanomas.

#### Casein kinase II (CKII)

Translocation t(9:22), which codes for BCR-ABL chimeric protein, causes Chronic Myeloid Leukaemia (CML).^159^ It was shown that BCR-ABL leads to the shuttling of PTEN from the nucleus to the cytoplasm and results in the loss of PTEN nuclear function.^160^ Morotti *et al*., 2015 demonstrated the mechanism of how PTEN is inactive in the cytoplasm, showing that BCR-ABL inactivates PTEN via the activity of CKII.^161^ The study highlighted a novel pathway, BCR-ABL/CKII/PTEN as a potential target for synthetic lethality by using a tyrosine kinase inhibitor.

#### Ataxia telangiectasia mutated (ATM)

*PTEN*-deficient cells increased the level of reactive oxygen species, and endogenous DNA damage and activate ATM molecule that has a role in the DNA damage response.^162^ The synthetic lethal interaction between PTEN and ATM was identified and inhibition of ATM leads to catastrophic DNA damage, mitotic cell cycle arrest, and cell death in *PTEN*-deficient cells. This finding suggested that the survival of *PTEN*-deficient can depend on ATM activation to maintain the integrity of DNA.^162^ A different study also showed that PTEN and ATM are synthetic lethal partners in breast cancer cells and demonstrated that the sensitivity of *PTEN*-deficient breast cancer cells to cisplatin was increased with ATM inhibitor KU-60019.^163^

#### Death domain-associated protein (DAXX)

As PTEN physically interacts with DAXX and regulates the loading of H3.3 on chromatin in GBM, the chromatin-associated role of PTEN was discovered. This interaction between PTEN and DAXX-H3.3 chromatin complex represses the transcription of oncogenes.^164^ Therefore, in *PTEN*-deficient tumour cell, H3.3 is removed from the chromatin by DAXX and increases the expression of the oncogene. Inhibition of *DAXX* restored H3.3 on the chromatin, inhibited the level of oncogenes, suppressed the growth of tumour cells, and improved the survival of *PTEN*-deficient GBM cells in a mice model. This study highlighted the synthetic lethal interaction between PTEN and DAXX in GBM.^164^

#### Chromatin helicase DNA binding protein 1 (CHD1)

Another study discovered *CHD1* as a synthetic essential gene in *PTEN*-deficient cancers.^165^ Cell proliferation, survival and tumorigenic potential were suppressed with the inhibition of *CHD1* in *PTEN*-deficient cancer breast and prostate cancers. Mechanistically, PTEN inhibits AKT, which then activates GSK3β. Activated GSK3β phosphorylates and degrades CHD1 via a β-TrCP mediated ubiquitination-proteasome pathway. In contrast, *PTEN*-deficient prostate cancer cells stabilise CHD1 protein and lead to its interaction with H3K4me3 and transcriptional activation of NF-κB downstream genes to cause the progression of prostate cancer. Additionally, inhibition of *CHD1* suppresses the proliferation and tumour growth of both prostate and breast cancer cells with *PTEN* deficiency. This study demonstrated a novel pathway of PTEN in cancer and suggested potential targeted therapy for *PTEN*-deficient tumours.

#### Dihydroorotate dehydrogenase (DHODH)

Although the role of PTEN in glucose metabolism is not completely understood, and one of the studies examined the metabolic consequences of PTEN loss. It was found that glutamine flux increased the growth of *PTEN*-inactive cells via the de novo pyrimidine synthesis pathway and this increased the sensitivity to DHODH enzyme inhibition.^166^ The number of replication forks was increased in PTEN-mutant cells that are in the S-phase of the cell cycle and suppression of DHODH caused chromosome breaks and apoptosis due to the impotent activation of ATR and DNA damage at replication forks.^166^ Therefore, this study discovered that glutamine flux increased the sensitivity to DHODH suppression which leads to synthetic lethality in *PTEN*-deficient cells, and suggested DHODH could be a potential therapy for *PTEN*-deficient cancer patients. Recently, it was shown that by using a DHODH inhibitor, leflunomide synthetic lethality in *PTEN*-deficient prostate cancer was triggered both in vitro and in vivo.^167^

#### NUAK family kinase 1 (NUAK1)

By using a multi-step approach; (1) siRNA screen in isogenic human mammary epithelial cell lines, (2) shRNA screen in breast cancer cell lines, (3) identifying hits between siRNA-shRNA screens and 3 independent gene essentiality screens, and (4) drug sensitivity assay in cell lines or publicly available pan-cancer somatic mutation data, *PTEN* synthetic lethal genes were identified in breast cancer. One of the identified novel *PTEN* synthetic genes was *NUAK1* and it was shown that *NUAK1* inhibition by small molecule drug HTH-01-015 decreased the viability of *PTEN*-deficient breast cancer cell lines.^168^ This study also highlighted a potential treatment for *PTEN*-deficient breast tumours.

#### Ataxia telangiectasia-mutated- and Rad3-related kinase (ATR)

The protein level of ATR was examined in human breast cancers and it was found that ATR level was highly expressed in low nuclear PTEN tumours, which was associated with higher grade, larger tumour size, and poor survival.^169^ ATR was blocked with VE-821 which led to double-strand DNA breaks, cell cycle arrest, and an increase in apoptosis.^169^ This study demonstrated the synthetic lethality relation between *PTEN*-deficient triple-negative breast cancer and ATR.

#### Pyruvate dehydrogenase kinase 1 (PDHK1)

Chatterjee *et al*., 2019 showed that metabolic PDHK1 expression was upregulated in *PTEN*-deficient lung adenocarcinoma.^170^ It was also found that inhibition of *PDHK1* by shRNA and PDHK1 inhibitor dichloroacetate (DCA) in *PTEN*-deficient cancer cells led to synthetic lethality. Mechanistically, it was shown that loss of PTEN protein-phosphatase activity phosphorylates NKAP, NF-κB activation and PDHK1 upregulation. Upregulation of PDHK1 promotes aerobic glycosylation, suggesting that the NKAP and PDHK1 are important for the survival of PTEN protein-phosphatase deficient cells.^170^ This study identified PDHK1 as a potential targeted therapy for *PTEN*-deficient cancers.

#### Lysyl oxidase (LOX)

The combination of profiling and functional studies in GBM demonstrated that loss of *PTEN* increases macrophage infiltration through the activation of the YAP1-LOX-β1 integrin-PYK2 pathway and the survival of GBM is sustained by the secretion of SPP1 from infiltrated macrophages.^171^ Macrophage infiltration and tumour growth were decreased with the inhibition of *LOX* in GBM xenograft mouse models.^171^ This study showed the interaction and mechanism between glioma cells and macrophage, which revealed a potential therapeutic target for *PTEN*-deficient GBM.

#### WD repeat and HMG-box DNA binding protein 1 (WDHD1)

**Figure 6:**
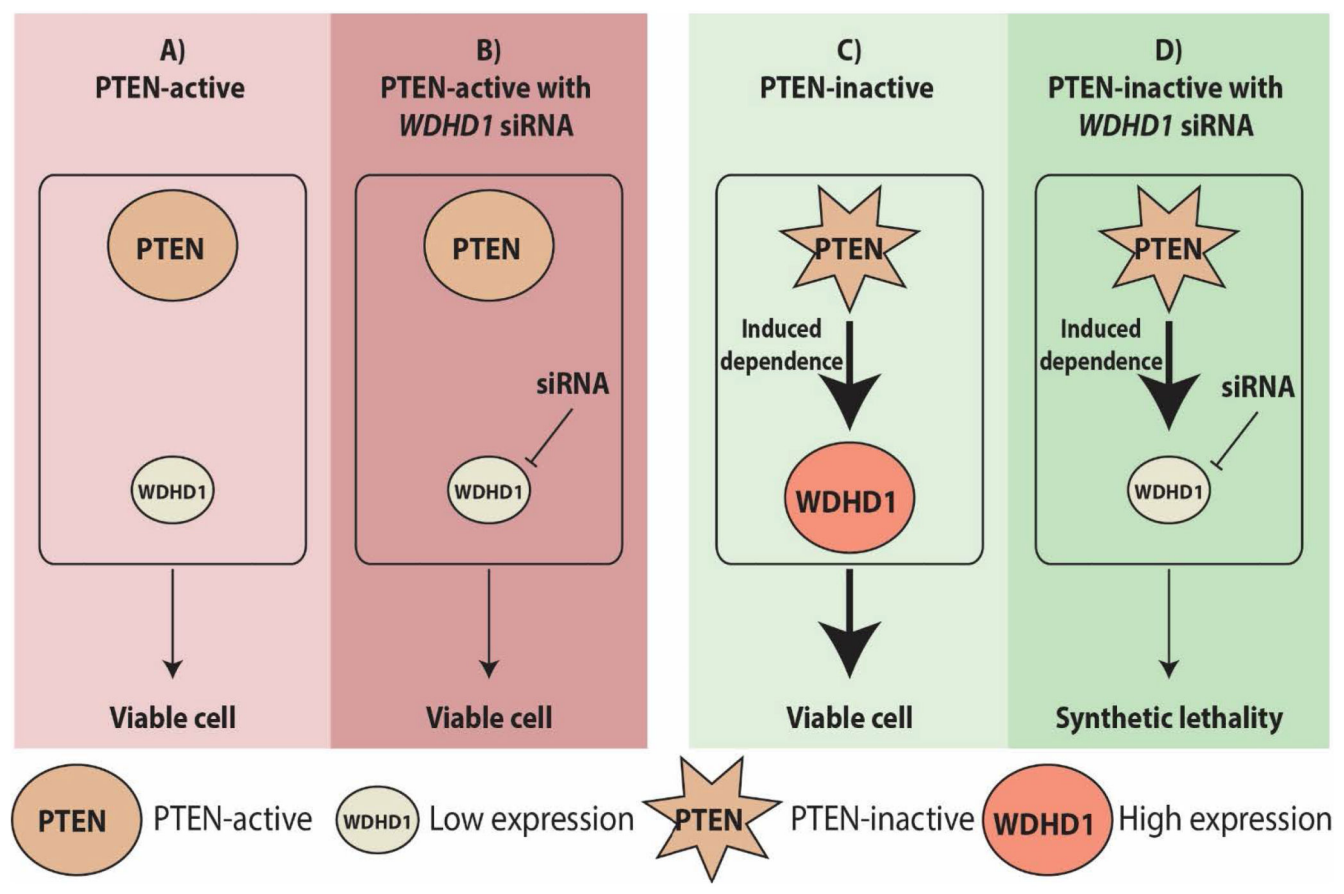
*WDHD1* as a synthetic essential gene in PTEN-inactive TNBC cells. **A)** *WDHD1* expression is low in PTEN-active TNBC. **B)** Knockdown of *WDHD1* with siRNA does not decrease the cell survival in PTEN-active TNBC cells. **C)** PTEN-inactive TNBC cells increase *WDHD1* expression and the survival of cells. **D)** Inhibition of *WDHD1* with siRNA in PTEN-inactive TNBC leads to synthetic lethality (cell death). The star represents the inactive PTEN. Arrows indicate induction. Bold arrows indicate higher induction. PTEN: phosphatase and tensin homolog; WDHD1: WD repeat and high mobility group [HMG]-box DNA binding protein 1; TNBC: triple negative breast cancer.

In our recent study, we conducted a joint analysis using TCGA data and whole genome siRNA screening in isogenic PTEN-negative and -positive cells to discover PTEN synthetic lethal genes.^172^ *WDHD1* was one of the identified candidate synthetic essential genes in PTEN-inactive TNBC cells (Fig. 6). Among the candidate genes essential for the survival of PTEN-inactive TNBC cells, WDHD1 expression was higher in *PTEN*-low TNBC samples compared to the *PTEN*-high TNBC samples. siRNA screening also showed that *WDHD1* was the top hit gene and the cell viability of PTEN-negative cells was significantly inhibited with the knockdown of *WDHD1*, which was further validated in 2D and 3D cultures.^172^ We also showed that the expression of WDHD1 in TNBC is affected by PTEN status via AKT signalling. Patient samples obtained from the TCGA and tissue microarrays with clinic-pathological information also supported the significance of WDHD1 in TNBC. Mechanistically, WDHD1 plays an important role in cell cycle progression as well as mediating a high demand of protein translation in PTEN-inactive TNBC via directly interacting with the components of the translation machinery. Thus, as an essential gene for the survival of PTEN-inactive TNBC cells, WDHD1 could be a potential therapeutic target for TNBC.

#### Histone Acetyltransferase (HAT) P300/CBP

Synthetic lethality drug screening with PTEN-isogenic colorectal cancer cells discovered that *PTEN*-deficient cells were sensitive to anacardic acid, a p300/CBP HAT inhibitor.^173^ Cell viability of *PTEN*-deficient cells was decreased with anacardic acid due to the induction of apoptosis. Anacardic acid reduced the acetylation of histones and downregulated the Hsp70 family of proteins transcription, which decreased the formation of the AKT-Hsp70 complex and phosphorylation of AKT at Ser473. The validation of the synthetic lethality of anacardic acid in *PTEN*-deficient tumours was performed in vivo.

## Conclusion

Understanding signalling pathways in cancer is very important for the effect and response of a potential drug. Inactive tumour suppressor genes can alter the downstream signalling pathways. Therefore, targeting the downstream signalling pathway, synthetic lethality, is an alternative approach to treat cancers with inactive tumour suppressor genes.

Clinically, the biggest limitation of synthetic lethality is drug resistance.^174,175^ Moreover, synthetic lethality interactions could be cancer-specific which means while it is successful in one cancer, it is unsuccessful in a different cancer. Thus, specific internal and external requirements are needed for the effect of synthetic lethality.^176^ To overcome the problems of drug resistance and the specific requirements of the cancer types, a multi-faceted testing framework could be used.^177^ In preclinical studies, drug resistance mechanisms can be observed when distinct microenvironments and genetic backgrounds of cancer cells are discovered which leads to different sensitivities to the same synthetic lethal effect.^178^ To solve the drug resistance mechanism and also reduce drug dosage, chemotherapeutic drugs, immunotherapy, or radiation therapy could be combined with a synthetic lethality-based treatment strategy.^174^ Synthetic lethal drugs may also have off-target side effects, increase the side effects of anticancer drugs, and damage DNA on normal tissue which may result in secondary malignancies.^144^ Nanomedicine has been a promising tool for effective drug delivery; to prevent adverse events, off-target side effects, and usage of high drug dosage.^179–181^ Therefore, integrating nanomedicine into synthetic lethality has the potential to overcome the limitations of synthetic lethality and also to improve the efficiency of the treatment.^182–184^ Synthetic lethality helped to provide different possibilities for the applications that are used at present and will be used in the future.

*PTEN* is the second most mutated tumour suppressor gene after *TP53* and the deficiency of PTEN was observed in different cancer types.^5^ Identifying a targeted synthetic lethal gene for *PTEN*-deficient cancer cells, might be used as a biomarker for treatment. However, as cancer is a heterogeneous disease, it is challenging to identify potential synthetic lethal genes, which may lead to identifying inaccurate biomarkers or targeted therapies. Therefore, large-scale high-throughput synthetic lethal screening approaches such as RNAi and CRISPR systems can be useful to discover synthetic lethal genes for cancer types with particular gene signatures such as PTEN deficiency. In this review, we uncover the importance of PTEN in cancer and synthetic lethality phenomena. Various studies showed the synthetic lethal interaction between the specific genes and PTEN, which could be a potential targeted therapy in cancer.

## Figures and Tables

**Table 1:** Identified synthetic lethality genes/interactions in PTEN-inactive cancer types.

Synthetic lethality genes/interactions	Function	Tumour cell line	Reference
*Poly-ADP ribose polymerase* (*PARP*)	DNA repair mechanism	Colorectal Endometroid Breast Glioma Bladder Melanoma	^150,152^
*MPS1, Mono Polar Spindle 1 (TTK)*	Regulates cell division	Breast	^153^
*Polo-like kinase 1 (PLK1)*	Regulates cell cycle	Prostate	^134^
*Nemo-like kinase* (*NLK)*	Regulates transcriptional molecules such as AKT-independent phosphorylation of FOXO1	Colorectal Endometrial Ovary Bladder Melanoma Lung Breast	^154^
Polynucleotide kinase-phosphatase (*PNKP)*	DNA repair mechanism	Lung Colon Prostate	^155,156^
*Apurinic/apyrimidinic endonuclease 1* (*APE1*)	DNA base excision repair (BER)	Melanoma	^158^
*Casein Kinase II* (*CKII*)	Cell cycle control DNA repair Cellular processes	Chronic myeloid leukaemia	^161^
*Ataxia telangiectasia mutated (ATM)*	DNA repair	Colorectal Prostate Breast	^162,163^
*Death domain associated protein* (*DAXX*)	Histone chaperone	Glioblastoma	^164^
*Chromatin helicase DNA binding protein 1* (*CHD1*)	Activate gene transcription	Prostate Breast	^165^
*Dihydroorotate dehydrogenase (DHODH)*	de novo pyrimidine synthesis	Breast Glioblastoma Prostate	^166,167^
*NUAK family kinase 1* (*NUAK1*)	Cell proliferation, cell cycle, DNA repair	Breast	^168^
*Ataxia telangiectasia-mutated- and Rad3-related kinase* (*ATR*)	DNA repair	Breast	^169^
*Pyruvate dehydrogenase kinase 1* (*PDHK1*)	Regulates energy metabolism	Lung	^170^
*Lysyl oxidase (LOX)*	Recruits macrophages	Glioblastoma	^171^
*WD Repeat And HMG-Box DNA Binding Protein 1 (WDHD1)*	Initiate DNA replication	Triple negative breast cancer	^172^
*Histone Acetyltransferase (HAT) P300/CBP*	Regulating gene transcription	Colorectal	^173^
